# Notes and News

**Published:** 1898-05-28

**Authors:** 


### NOTES AND NEWS.

(COLLECTED AND COMMUNICATED.)

MISS WINIFRED EMERY will give a matinée in aid of the funds of the Grosvenor Hospital for Women and Children at the Haymarket Theatre on Thursday, June 23rd, at 2.30 p.m. A most interesting programme has been arranged.

IT will be of interest to our readers and correspondents to know that the registered telegraphic address of the Scientific Press is "OSPEDALE, LONDON." These two words will in future be all that will be required in addressing telegrams either to THE HOSPITAL or THE MIRROR or THE SCIENTIFIC PRESS from any part of the United Kingdom, and with the addition of the word England from any part of the world.

A DEPUTATION, headed by Sir Charles Dilke, recently waited on the Home Secretary, asking that a woman inspector should be appointed for the Potteries, to supervise the young women working in china factories where lead is largely employed in glaze, &c. The Government report shows an average of 400 cases yearly from lead poisoning in the Potteries. Sir M. White Ridley could not promise the appointment of a female inspector, but Sir C. Dilke said nothing short of this would satisfy the North Staffordshire Trades Council and other organised bodies of workers in the county.

THE appointment of the Royal Commission on the Disposal and Treatment of Sewage, the constitution of which was recently announced by Mr. Chaplin, has come none too soon, for the question between the chemical and the bacteriological treatments of sewage is rapidly becoming acute. Even between the advocates of the various forms of biological treatment questions are pending on the solution of which hinge many disposal schemes involving the expenditure of very large sums of money. Manchester and Salford are cases in point, and at the latter place very earnest protests have been raised against the action of the Local Government Board, requiring the large amount of land to be devoted to sewage treatment which is demanded by the older and officially recognised methods of irrigation or land filtration. The position of Manchester is both serious and peculiar. On the one hand penalties are hanging over it for every day that it discharges unpurified sewage; and, on the other hand, the very appointment of this Royal Commission shows the doubt which hangs over the question whether the scheme on which the city is asked to spend a great sum of money is the best that can be adopted.

AT the Royal Free Hospital dinner at the Hotel Cecil, at which the Marquis of Dufferin strongly advocated the medical education of women, donations of £3,000 were announced. To carry out the necessary improvements in the wards, nurses' quarters, heating, and the provision of lifts, £10,000 is required.

COLLEAGUES, friends, and pupils of Sir William Stokes celebrated the silver jubilee of his professorship in surgery at a pleasant meeting held at the Royal College of Surgeons, Dublin. Accompanying an address were gifts of a pair of silver candelabra, a pair of large richly-chased silver bowls, and an exquisite diamond star for Lady Stokes.

MRS. MOLYNEUX, the Australian actress, is arranging a matinée on June 9th in aid of the Convalescent Home for Children in connection with Charing Cross Hospital. Mr. Gatti has kindly given the Vaudeville Theatre for the occasion; the stage hands are also offering their services. The programme promises to be both interesting and amusing.

THE Provost of the Guild of St. Luke and Mrs. Symes-Thompson gave an "At Home" on Wednesday night, May 25th, to meet the Bishops of Wakefield, Bristol, Rochester, and the Falkland Islands. The drawing room was crowded, and some very interesting addresses on the subject of Medical Missions were given by the Bishops of Wakefield and Bristol.

THE Duke of Westminster, presiding at a meeting of the governing body of the Belgrave Hospital for Children, held to discuss the proposed removal to Kennington, stated that the South of London badly needed a children's hospital, as shown by the fact that in the special hospitals for children in the south there is only one cot to 12,650 of the population, while on the north side there is one cot to 3,523.

THE Press Bazaar in aid of the London Hospital, which will be opened at the Hotel Cecil on June 28th and 29th by H.R.H. the Princess of Wales, is likely to be a brilliant affair. THE HOSPITAL will have a stall at which fruit will be sold, and we shall be pleased to receive gifts from our friends, and from any who wish well to the London Hospital, so as to render this stall as attractive as possible, nor need such gifts be confined to fruit. A certain latitude is always allowable at bazaars, and we have no doubt that we shall be able to find purchasers for all sorts of nice things sent in so good a cause.

UPON the resignation of Dr. Greene as medical superintendent of the County Asylum at Berry Wood —after holding the position for a period of twenty years—it was decided by the Committees of Visitors to make some small acknowledgment of the excellent services he had rendered the county in that capacity, and an elaborately illuminated address of a costly description, bound in morocco leather, has been presented to him. The address expresses the regret of the members of the Committees of Visitors at Dr. Greene's resignation, and a grateful acknowledgement of the services rendered by him to the county of Northampton, and of his kindness and courtesy to all with whom he has been brought in contact.

